# Marked reductions in outpatient antibiotic prescriptions for children and adolescents – a population-based study covering 83% of the paediatric population, Germany, 2010 to 2018

**DOI:** 10.2807/1560-7917.ES.2020.25.31.1900599

**Published:** 2020-08-06

**Authors:** Jakob Holstiege, Maike Schulz, Manas K Akmatov, Annika Steffen, Jörg Bätzing

**Affiliations:** 1 Central Research Institute of Ambulatory Health Care in Germany (ZI), Berlin, Germany

**Keywords:** antibiotics, children, Germany, infectious diseases, practice guidelines, outpatients, prescription rates, regional variation, statutory health insurance, surveillance, trend analysis

## Abstract

**Background:**

Prescribing of systemic antibiotics in general and of cephalosporins in particular in German paediatric outpatients has previously been reported to be higher than in other European countries.

**Aim:**

Our objective was to assess recent trends in antibiotic prescribing in German children.

**Methods:**

This study was conducted as consecutive annual cross-sectional analyses and included all children aged 0–14 years (n = 9,389,183 in 2018) covered by statutory health insurance in Germany. Annual antibiotic prescription rates from 2010 to 2018 were calculated for the age groups 0–1, 2–5, 6–9 and 10–14 years. Poisson regression was used to estimate trends of prescription rates by age group and antibiotic subgroup.

**Results:**

Overall, the age-standardised antibiotic prescription rate decreased significantly by 43% from 746 prescriptions per 1,000 persons in 2010 to 428 per 1,000 in 2018 (p < 0.001). Reductions were most pronounced in the age groups 0–1 year (−50%) and 2–5 years (−44%). The age group 2–5 years exhibited the highest prescription rate with 683 per 1,000 in 2018 (0–1 year: 320/1,000; 6–9 years: 417/1,000; 10–14 years: 273/1,000). Cephalosporins (second and third generation) accounted for 32% of prescribed antibiotics.

**Conclusions:**

Marked reductions in antibiotic prescribing during the last decade indicate a change towards more judicious paediatric prescribing habits. Compared with other European countries, however, prescribing of second- and third-generation cephalosporins remains high in Germany, suggesting frequent first-line use of these substances for common respiratory infections. Considerable regional variations underline the need for regionally targeted interventions.

## Introduction

Antibiotic resistance is considered an emerging threat to global public health. Among other factors, exposure to antibiotics is a leading cause for the proliferation of resistant bacterial strains on individual [1] and population level [2]. Because the burden of respiratory infections in paediatric populations is high, outpatient antibiotic prescribing is particularly common in the treatment of childhood diseases. Many common respiratory infections, however, are predominantly caused by viruses and rarely benefit from antibiotic treatment, implying an unfavourable risk–benefit ratio of antibiotic treatment in many cases [3,4]. Hence, high outpatient antibiotic prescribing to paediatric populations is a recognised indicator for inappropriate prescribing patterns [5].

Data from 2008 illustrated wide variability of outpatient paediatric antibiotic prescribing patterns in European countries [6]. With a prescription rate of 561 prescriptions per 1,000 person-years, antibiotic use among German children and adolescents aged 0–18 years was higher than corresponding figures reported for Denmark (481/1,000) and the Netherlands (294/1,000). Of particular concern were the high prescription rates of cephalosporins, suggesting common usage of these substances as first-line treatment of childhood infections in the German paediatric outpatient setting. This is in conflict with evidence-based practice guidelines for common childhood infections [7,8] and may further accelerate the emergence of antibiotic resistance among Gram-negative bacteria, including the selection of extended-spectrum beta-lactamases (ESBL) [9].

For the years 2010 to 2014, a study based on German nationwide data illustrated a strong decline in outpatient antibiotic use among children and adolescents in the age group 0–14 years by ca 30% [10]. So far, developments in later years have not been assessed and trends among different paediatric age groups are unknown. The present study aimed to assess the trends in outpatient antibiotic prescribing to German children and adolescents between 2010 and 2018, giving special attention to age group-specific prescribing, regional variations and use of different antibiotic subgroups.

## Methods

### Study data and population

Our study was designed as consecutive annual cross-sectional analyses based on nationwide outpatient prescription data of all children and adolescents aged 0–14 years covered by statutory health insurance (SHI) in the years 2010 to 2018. These data cover ca 83% of the German population in this age group and comprise all prescribed and dispensed ambulatory medications (except for dental prescriptions), in accordance with §300 paragraph 2 SGB (German Social Security Code) V. They include the patient’s age, the date of prescription, the pharmacy dispensation date, the amount of the prescribed substance, the anatomical therapeutic chemical (ATC) code, the defined daily doses (DDD), packaging size, strength and formulation as well as the generic and trade names. The study authors had unrestricted access to the database used. 

The study population was divided into the age groups 0–1, 2–5, 6–9 and 10–14 years, following recommendations for age categorisation in paediatric clinical studies [11]. The total annual number of insured persons per age group and federal state recorded on 30 June of a given year was used as reference population, derived from national statistics provided by the German Ministry of Health [12]. Since this reference population merely provides the size of the total SHI population aged 0–14 years in each federal state, we estimated the size of each SHI age group assuming the same proportions as in the population of each federal state.

### Antibiotic subgroups and utilisation measures

Outpatient prescriptions of systemic antibiotics (ATC code: J01) were divided into the following subgroups: broad-spectrum penicillins (J01CA), narrow-spectrum penicillins (J01CE, J01CF), penicillins with beta-lactamase inhibitors (J01CR), first-generation cephalosporins (J01DB), second-generation cephalosporins (J01DC), third-generation cephalosporins (J01DD), sulphonamides/trimethoprim (J01EB, J01EE, and J01EA) and macrolides (J01FA). Less frequent antibiotics were pooled in the subgroup ‘others’. Use of systemic antibiotics was measured as the annual prescription rate, i.e. the number of prescriptions per 1,000 persons per year, in total and by age group, federal state as well as antibiotic subgroup. As dosage of antibiotics depends on a patient’s age and body weight, prescription rates are more appropriate to describe antibiotic use among children and adolescents in different age groups than DDD per population [5].

A study of outpatient antibiotic prescriptions from the Netherlands showed that the vast majority of antibiotics are prescribed as single course [13]. We assume a similar prescribing pattern in German outpatient care. Hence, the prescription rate is a good approximation for the rate of antibiotic treatments at population level. In addition, prescription rates are robust towards alterations of dosage per prescription over time, which may arise from changes in the prescribing practice for specific drugs or changes in the mix of antibiotics used [14]. To capture possible changes in dosage per prescription over time, mean annual DDD per prescription were estimated.

### Trend analyses

We estimated the annual percentage change in prescription rates using Poisson regression. The analysis was based on monthly prescription rates and models were run separately for each age group and antibiotic subgroup. Season was included as a dummy-coded variable with four categories, one for each quarter of a year (reference period: third quarter, i.e. the summer quarter). We assumed that there was a log-linear relationship between the year and the prescription rate. Rate ratio (RR) estimates for calendar year and season as well as corresponding likelihood ratio test p values were calculated. As calendar year was included as continuous variable, RR-1 corresponded to the mean estimated relative change in prescription rate from one year to the next. All models incorporated a dispersion parameter to avoid overdispersion. Statistical analyses were conducted using SAS 9.4. 

### Ethical statement

In Germany, the use of data from insurance claims for scientific research is regulated by the Code of Social Law (SGB X). Ethical approval and informed consent were not required as our study used routinely collected anonymised data.

## Results

In 2018, the study population comprised 1,328,589 (14%) children in the age group 0–1 year, 2,520,176 (27%) in the age group 2–5 years, 2,446,918 (26%) in the age group 6–9 years and 3,093,500 (33%) children and adolescents in the age group 10–14 years. Between 2010 and 2018, the size of the population aged 0–14 years increased by 3% from 9,132,142 to 9,389,183.

**Figure 1:**
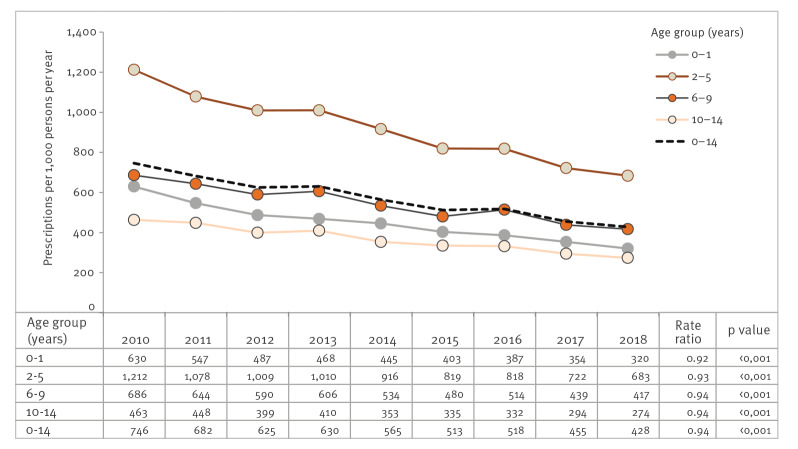
Age-standardised prescription rates of systemic antibiotics and age group-specific rate ratios of antibiotic prescription rates, Germany, 2010–2018

Between 2010 and 2018, the age-standardised antibiotic prescription rate in children and adolescents (0–14 years) decreased by 43% from 746 (2010) to 428 (2018) prescriptions per 1,000 persons per year (Figure 1), corresponding to an average annual decrease of 6% (RR: 0.94; p < 0.001). The downward trend of the crude prescription rate was slightly less pronounced compared with the age-standardised prescription rate (−41%; 2010: 731/1,000; 2018: 428/1,000) (Table). The mean number of DDD per prescription changed marginally during the study period (2010: 7.9; 2018: 7.4).

Prescription rates for the total study population 0–14 years were standardised according to age and sex taking as reference the paediatric population covered by statutory health insurance in 2010. They are shown underneath the figure as rates per 1,000 persons.

**Table:** Crude antibiotic prescription rates in 2010 and 2018 and rate ratio estimates from Poisson regression for the years 2010−2018 of total and subgroup-specific systemic antibiotic prescription rates, for change per year and different seasons compared to summer months, Germany

Antibiotic subgroup	Crude prescription rate		Annual change 2010−2018		Seasonal relative change compared with third quarter					
					First quarter		Second quarter		Fourth quarter	
	2010	2018	Rate ratio	p value	Rate ratio	p value	Rate ratio	p value	Rate ratio	p value
Narrow-spectrum penicillins	95.2	69.1	0.97	< 0.001	1.62	< 0.001	1.31	< 0.001	1.33	< 0.001
Broad-spectrum penicillins	166.4	121.2	0.96	< 0.001	2.28	< 0.001	1.26	< 0.001	1.70	< 0.001
Penicillins with beta-lactamase inhibitor	16.0	16.7	1.01	0.245	1.54	< 0.001	1.11	0.006	1.27	< 0.001
First-generation cephalosporins	16.9	6.1	0.88	< 0.001	1.71	< 0.001	1.33	< 0.001	1.28	< 0.001
Second-generation cephalosporins	186.7	115.5	0.95	< 0.001	1.83	< 0.001	1.16	< 0.001	1.41	< 0.001
Third-generation cephalosporins	61.9	22.3	0.87	< 0.001	2.06	< 0.001	1.25	< 0.001	1.54	< 0.001
Macrolides	143.9	52.2	0.88	< 0.001	2.34	< 0.001	1.16	0.025	1.80	< 0.001
Sulfonamides/trimethoprim	30.7	16.1	0.92	< 0.001	1.32	< 0.001	1.04	0.101	1.21	< 0.001
Others	13.3	8.3	0.94	< 0.001	1.00	0.991	0.94	0.001	1.16	< 0.001
**All**	**731.0**	**427.5**	**0.94**	**< 0.001**	**1.93**	**< 0.001**	**1.20**	**< 0.001**	**1.51**	**< 0.001**

Results from Poisson regression analyses represent changes from one year to another over the study period (2010−2018) and changes between seasons (reference period: summer, 3rd quarter) of antibiotic prescription rates.

The highest percentage decrease in the prescription rate by almost half (−49%; RR: 0.92; p < 0.001) was observed in the age group 0–1 year (2010: 630/1,000; 2018: 320/1,000). On the absolute scale, 2–5 year-olds showed the highest reduction in the prescription rate with 530 fewer prescriptions per 1,000 persons in 2018 (683/1,000) compared with 2010 (1,213/1,000), corresponding to a percentage decrease of −44% (−RR: 0.93; p < 0.001) (Figure 1). Nevertheless, with 683 prescriptions per 1,000 persons, the age group 2–5 years still exhibited by far the highest prescription rate in 2018 (0–1 year: 320/1,000; 6–9 years: 417/1,000; 10–14 years: 273/1,000) (Figure 1).

**Figure 2:**
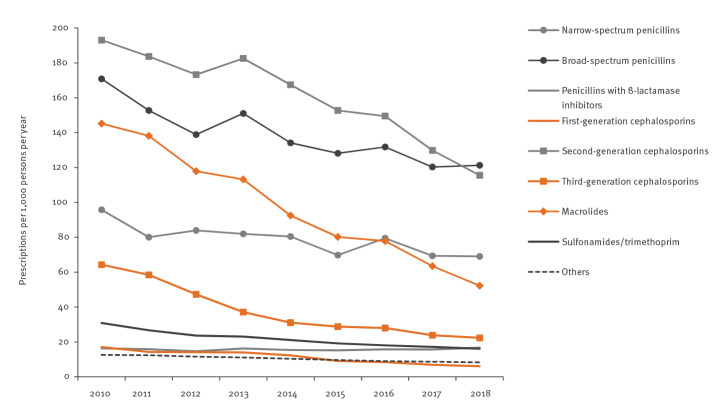
Age-standardised prescription rates per 1,000 persons per year of systemic antibiotic subgroups in 0–14 year-olds, Germany, 2010–2018

With the exception of penicillins with beta-lactamase inhibitor, prescription rates of all antibiotic subgroups decreased markedly. Relative reductions varied between −64% (macrolides (RR: 0.88) and third-generation cephalosporins (RR: 0.88)) and −27% (broad-spectrum penicillins (RR: 0.96) and narrow-spectrum penicillins (RR: 0.97) (Table and Figure 2). The vast majority of antibiotic subgroups (exception: others) showed higher prescribing in the first, second and fourth quarters compared with the third (summer) quarter (Table). The strongest seasonal variations measured by the RR for the first vs the third quarter could be observed for macrolides (RR: 2.34), broad-spectrum penicillins (RR: 2.28) and second-generation cephalosporins (RR: 1.83), corresponding to, respectively, 134%, 128% and 83% higher prescription rates in the first compared with the third quarter (Table).

Prescription rates were standardised according to age and sex using as reference the paediatric population covered by statutory health insurance in 2010.

**Figure 3:**
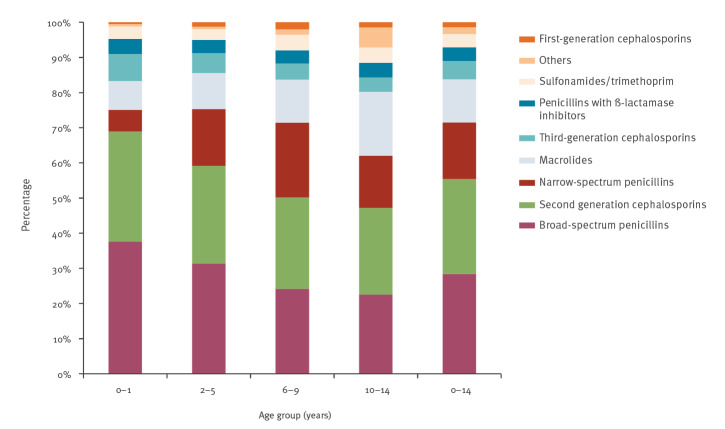
Distribution of systemic antibiotic subgroups by age group (0–14 year-olds) as percentages of the total antibiotic prescriptions per age group, Germany, 2018 (n = 9,389,183)

In 2018, broad-spectrum penicillins and second-generation cephalosporins were the most dispensed antibiotic subgroups in all age groups, amounting to 55% of antibiotics prescribed for German children (broad-spectrum penicillins: 28%, second-generation cephalosporins: 27%) (Figure 3). For these two subgroups as well as third-generation cephalosporins, the proportions in relation to all prescriptions gradually decreased with increasing age. In contrast, this pattern was reversed for macrolides, which showed the highest percentage in the age group 10–14 years (18%) and the lowest among very young children (0–1 year: 8%). The subgroup ‘others’ was mainly used in children of 10–14 years, representing 5.7% of prescribed antibiotics in this age group (Figure 3).

**Figure 4:**
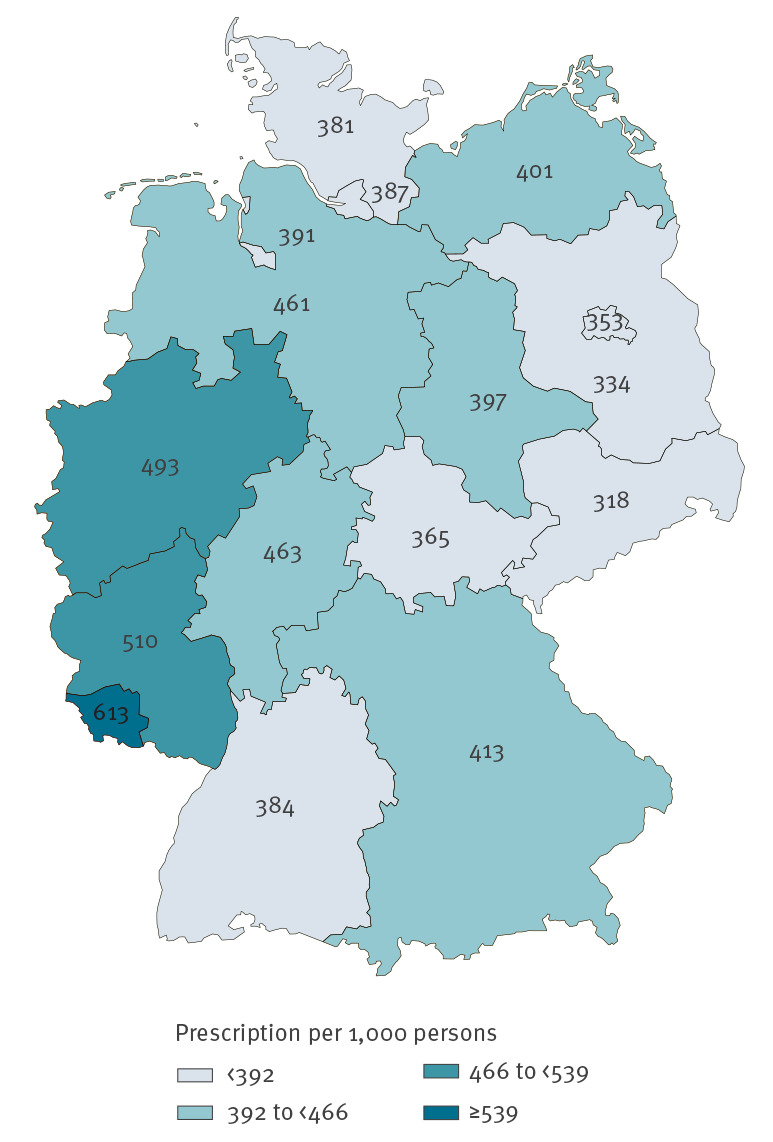
Age-standardised systemic antibiotic prescription rates per 1,000 persons, by federal state (0–14 year-olds), Germany, 2018 (n = 9,389,183)

The regional distribution of prescription rates revealed a cluster of high paediatric use in western Germany comprising the federal states North Rhine-Westphalia, Rhineland-Palatinate and Saarland (Figure 4). Over the course of the study, the coefficient of variation in the prescription rates between federal states increased from 14% (2010) to 18% (2018), corresponding to a rise in the regional variation by 29%. In 2018, antibiotic use varied by a factor of 1.9, with Saxony (318/1,000) showing the lowest and Saarland (613/1,000) the highest prescription rate (Figure 4).

Prescription rates were standardised according to age and sex taking as reference the paediatric population covered by statutory health insurance in 2010.

## Discussion

Our study on recent trends in antibiotic prescribing in the outpatient paediatric setting in Germany showed marked reductions in prescription rates over the last decade. The rates declined strongly in all regions, for the vast majority of antibiotic subgroups and for all paediatric age groups, but the relative reductions were most pronounced in very young children (0–1 year-old).

In Europe, country-specific outpatient antibiotic use in the total population is continuously monitored and published in annual reports by the European Surveillance of Antimicrobial Consumption Network (ESAC-Net), repeatedly showing considerable variations in prescribing across countries [15]. These data allow comprehensive comparisons between the overall levels of outpatient antibiotic use and regularly rank Germany in the quintile of countries with the lowest antibiotic use. Nevertheless, data from ESAC-Net do not allow comparing age group-specific prescribing patterns. Such comparisons are only conducted sporadically, i.e. in single studies mostly comparing two countries [5,16-18] and a cohort study comprising five European countries [6]. Despite the low use of antibiotics in the total German population, prescription rates in German children were markedly higher in that cohort study compared with their Danish, British and Dutch peers in 2008 [6]. Our study confirmed an unchanged high level of paediatric prescribing in the year 2010. After a stepwise decline in the prescription rate in the following 8 years, the overall reduction was 43%, suggesting a sustainable change in paediatric prescribing patterns. Strong improvements have also been observed in other European countries, including the Italian Region Emilia Romagna, the Netherlands, Scotland and Sweden. In Scotland, the antibiotic prescription rate in children younger than 5 years decreased by 60% from 1,612 to 634 per 1,000 persons between 1995 and 2014 [14]. In the Netherlands, antibiotic use among 0–14 year-olds, measured as packages per population, decreased by 8–17% depending on age between 2012 and 2016. Similar to German children, Dutch 0–1 year-olds exhibited the strongest decrease from 425 to 353 packages per 1,000 children per year [17]. In both countries, children aged 2–5 years showed the highest age group-specific use, most likely because of exposure to respiratory pathogens in daycare. In 2017, 90% of German 3–5 year-old children attended daycare centres [19].

In Germany, a considerable downward trend in paediatric antibiotic use may have been triggered by rising public awareness regarding the threat of antibiotic resistance, accompanied by increasing media coverage and an increasingly critical view of the use of antibiotics [16]. In Eurobarometer reports covering 27 European Union countries, Germany showed the strongest increase in percentage points for study participants who correctly answered four questions regarding antimicrobial resistance, from 15% in 2009 [20] to 30% in 2018 [21]. Furthermore, the German Antimicrobial Resistance Strategy ‘DART 2020’ to initiate and strengthen activities tackling the emergence of antibiotic resistance on national level was first introduced in 2008 and finalised in 2015 [22]. This led to several interventions promoting prudent antibiotic use in the community, targeting physicians, patients and the general public [23-25]. However, since the majority of these interventions did not start before 2016, the downward trend already observed in earlier years cannot be attributed to these interventions. 

Other factors include an increasing focus on judicious prescribing habits in continuous medical training of German physicians and the release of two national guidelines for medical practice, one by the National Association of Statutory Health Insurance Physicians in 2012 for the management of respiratory infections in general [8] and one by the German College of General Practitioners and Family Physicians on the management of sore throat in 2009 [26]. The latter may have been of particular importance as tonsillitis has been identified as the most important indication for outpatient antibiotic treatment of German children younger than 15 years in 2006 [27].

Finally, the introduction of pneumococcal vaccination in Germany in 2006 is likely to have contributed to the decrease in paediatric prescription rates as vaccination uptake increased strongly during the years of our study [28]. Pneumococcal vaccination has been associated with a marked drop in the incidence of acute otitis media and pneumonia in children in Iceland and a reduction in outpatient paediatric antibiotic prescriptions in randomised controlled trials and at least one observational study [29-31]. Following recommendations by the German Standing Committee on Vaccination to immunise all children until the 24th month of age with 7-valent pneumococcal vaccination in 2006 and, from 2009 onwards, with 10-valent and 13-valent vaccination [32], the proportion of children aged 5–7 years vaccinated against pneumococci increased from 14% in 2010 to 84% in 2017 [28].

The observed prescription rate in German children (0–14 years) of 428 prescriptions per 1,000 persons in 2018 was considerably lower than in Italian children in 2016 (881/1,000 [33]) and lower than the package rates from Hungary (1,386/1,000) and Portugal (838/1,000) in 2014, but more than 70% higher than package rates from Norway (236/1,000 in 2014) [18] and the Netherlands (247/1,000 in 2016) [17]. Of note, the package rates for Hungary, Portugal and Norway reported here for the age group 0–14 years were calculated based on the age-specific numerator and denominator data that can be found in Benko et al. [18], who included the age group 0–19 years in their study. The strong variations among European countries are unlikely to reflect an actual therapeutic need originating in marked inter-country differences in the burden of infectious diseases. However, because comprehensive studies on infectious disease aetiology and burden are lacking, differences in morbidity across European populations cannot entirely be ruled out. For young children (0–1 years-old), however, a multi-centre study has shown that the frequency of upper respiratory infections, acute otitis media, gastrointestinal infections and fever was comparable in Austria, Germany, Italy, the Netherlands and Switzerland [34]. Furthermore, a prospective study in 13 European countries revealed that differences in clinical presentation did not explain the considerable variation in antibiotic prescribing for acute cough [35].

Similar to Germany, considerable intra-country variation in paediatric prescribing has also been reported for Denmark, Slovenia and Italy [36-38]. Reasons for regional variation between German federal states have not been comprehensively studied but are unlikely to be justified by variations in the burden of infectious diseases. Future research should assess regional differences in attitudes and levels of knowledge in the community and among healthcare providers and may inform regionally tailored interventions to further promote judicious antibiotic prescribing.

Wide variability in Europe concerning the choice of antibiotic subgroups is well acknowledged for the paediatric setting as well as the total population [6,15]. In Germany, use of second- and third-generation cephalosporins was particularly high in young children, accounting for almost a third of all antibiotic prescriptions to German paediatric outpatients in 2018. Relative to penicillins, cephalosporins are most likely to increase the risk of *Clostridium difficile* infections in children [39] and are prone to accelerate the emergence of antibiotic resistance in Gram-negative bacteria, including the selection of ESBL [9]. 

In contrast, use of cephalosporins in children and the total population is negligible in Scandinavian countries, the Netherlands and the United Kingdom [6,17,18,37]. While German clinical practice guidelines for the outpatient treatment of acute respiratory infections commonly recommend cephalosporins as second- or third-line treatment [8,26], these antibiotics are not mentioned as treatment option by Dutch guidelines [17]. Further, the magnitude of cephalosporin prescription in German paediatric care suggests frequent utilisation of these substances as a first-line treatment for childhood respiratory conditions, thereby contradicting recommendations by German guidelines [8,26]. 

### Strengths and limitations

Our study provides a comprehensive analysis of recent trends in paediatric antibiotic prescription rates in Germany based on a full sample of nationwide outpatient prescription data of all individuals covered by SHI, i.e. 83% of German inhabitants in the age group 0–14 years. This contributes to ongoing surveillance of antibiotic use and allows healthcare practitioners and policymakers to audit outpatient prescribing patterns with regard to total and subgroup-specific antibiotic use in paediatric age groups and German regions.

Some limitations have to be taken into consideration. Firstly, as this study was based on antibiotics prescribed to the population covered by SHI, prescribing patterns in the population with private insurance, representing ca 13% of German inhabitants, remain unknown. Secondly, prescriptions issued by dentists were not included in the study. Antibiotic prescriptions by dentists account for ca 8–9% of outpatient antibiotic prescriptions for patients of all ages. Nevertheless, we assume that systemic antibiotic treatment by dentists is very rare in paediatric care. Finally, compliance with the dispensed antibiotic prescriptions cannot be assessed on the basis of claims data.

## Conclusions

As in other European countries, considerable reductions in antibiotic prescription rates during the last decade indicate a change towards more judicious patterns of antibiotic use in German outpatient paediatric care. However, as antibiotic use in German children is still ca 70% higher than among their Norwegian and Dutch peers, there is still room for further reduction. Intra-country variation of prescription rates underline the need for regionally targeted interventions. In addition, compared with other European countries, prescribing of second- and third-generation cephalosporins remains alarmingly high and suggests frequent first-line use of these substances in common respiratory infections, thereby contradicting recommendations by German guidelines.

## Data sharing statement

The datasets analysed during the current study are not publicly available due to data protection regulations by the German Social Security Code (Sozialgesetzbuch (SGB) V).
